# CD4+CD8+ Double-Positive T-Lymphocytes: Pitfalls

**DOI:** 10.4274/tjh.galenos.2020.2020.0140

**Published:** 2020-08-28

**Authors:** İrfan Yavaşoğlu

**Affiliations:** 1 Aydın Adnan Menderes University Faculty of Medicine, Division of Hematology, Aydın, Turkey

**Keywords:** CD4+CD8+ Double-positive T-lymphocytes, Pitfalls

## To the Editor,

The article entitled “Percentages of CD4+CD8+ Double-positive T Lymphocytes in the Peripheral Blood of Adults from a Blood Bank in Bogotá, Colombia,” written by Gonzalez-Mancera et al. [1] and published in a recent issue of your journal, was quite interesting. Herein, I wish to contribute to the article.

Nicotine has been reported to affect the cell-mediated immune system. In addition, nicotine exposure can lead to regulatory T-cell induction [2,3]. Therefore, I think that it is important to know the smoking status and also the number of lymphocytes for the subjects in Gonzalez-Mancera et al.’s  [1] study. Data have been published revealing that the prevalence of monoclonal B-cell lymphocytosis is higher than previously reported in blood donors [4]. Also, the large number of monoclonal B-cell lymphocytes determines the biological fate of cells transfused in recipients [4]. The use of CD45 during gating in flow cytometry could provide accurate identification. CD3+CD16/56 is important in determining natural killer T (NKT) cells and could have identified NKT cell contamination in the study.

Zloza and Al-Harthi reported that the expression of invariant and non-invariant NKT markers was most prominent in the CD4^bright^CD8^dim^ subpopulation [5]. In one study, up to 29% of cells were found as invariant CD3+6B11+NKT cells and up to 26% as non-invariant CD3+CD16/56+NKT cells [4]. It was also stated that the combination of cell populations not sharing similar features, expressing differentiation and activation of surface markers, and not consuming contaminating cell populations such as NKT cells would mask CD4+CD8+ T-cell subpopulation analyses [5].
